# Cases of Spinal Irritation, Producing and Modifying the Symptoms of Disease

**Published:** 1831

**Authors:** James C. Finley


### *Cases of Spinal Irritation, producing and modifying the symptoms of disease*—By James C. Finley, M. D.

In the two last numbers of the Journal, we presented our readers with a review of two works,* referring a great variety of diseases to spinal irritation, and pointing out a very simple mode of treating symptoms which have hitherto been not less perplexing to the physician, than painful and discouraging to the patient. To the class of diseases treated of by the first of these writers, our attention has been more particularly directed, on account of the greater frequency of these affections in ordinary practice. It will be recollected, that Dr. Tate, in the work referred to, asserts that hysteria, with all its varied symptoms, is invariably accompanied by derangement of the monthly evacuation, and by a tenderness of some one or more of the dorsal vertebræ; that by irritating the course of the spinal column, in the vicinity of the affected vertebræ, the distressing symptoms are generally alleviated, and that by a course of alo-

*Tate on Hysteria, and Teale on Neuralgic Diseases.

-etic purgatives, with such general treatment as the particular circumstances of the case may require, the disease is entirely relieved with the restoration of the healthy condition of this secretion.

Since this work came into our hands, we have had occasion to prescribe for an unusual number of cases of this description, and, indeed, we have been led to look for symptoms of hysteria, and deranged menstruation, in many cases where, otherwise, we should never have suspected it, and to prescribe successfully for symptoms under which females had labored for years, unpitied and unregarded. The result has been, that we have come to look upon the mode of treatment recommended by these gentlemen, as one of the most valuable suggestions that have been for a long time offered to the profession.

Few practitioners, we presume, are aware how frequently this secretion, upon which so much of the comfort and health of a female depends, is deranged. Still less are they conscious, how often an irregularity so slight as to escape the suspicion of the female herself, may produce consequences so serious as to embitter her whole life. Intelligent practitioners generally appear to have inclined to the opposite view of the subject, and to have considered these derangements rather as the consequence, than the cause of general disease; and although systematic works are burdened with remedies adapted to this condition, they have been very generally prescribed, rather to satisfy the anxiety of the patient or of her friends, than from any confidence in their virtues.

Although once inclined, with many others of our profession, to adopt this opinion, we cannot avoid thinking that the contrary sentiment, so universally entertained by the sex, is more consistent with the general laws of the animal economy.

Nature has given to those organs which are directly essential to the preservation of life, so delicate a sensibility, and such extensive sympathies, as at once to render them peculiarly liable to disease, and give them a controlling influence over the rest of the system. Now, no organ—not even the stomach, *the throne of life*, or the brain, the residence of the intellectual faculties—has, in this respect, been distinguished above the uterus. Not only does it impart to the woman, that physical organization, and moral and intellectual character, which prepare her to act her part in society, as the mother of her children; but although less exposed to direct causes of disease, than any other organ, it suffers perhaps, more than all the rest. This being the case, whenever it does become the seat of disease, whether it be primarily or secondarily affected, sound pathological views would expect it to become the *centre* of morbid associations, and to require a specific direction of our remedies.

Whether the remedies generally resorted to for this purpose, are calculated to effect their object, we are not qualified to decide, nor are we prepared to assign a reason for the efficacy of the one now under consideration. That it has the effect very generally we are convinced; and we feel no hesitation in asserting, that every case of hysteria, however aggravated, will be mitigated by it, and every recent case, if proper attention at the same time be paid to the primæviæ, will be promptly relieved. We shall take the liberty of presenting a few of the many cases, upon the result of which, our opinion of its efficacy, has been formed.

*Case I.* I was requested in June, 1831, to visit Mrs. A——, about 30 years of age, of full habits, and phlegmatic temperament. On my first visit, I found her laboring under the symptoms of bilious fever, combined with a severe affection of the chest. I was informed that about a week before, she had been visited by a physician, at this time absent from the city, who had bled and cupped her, and directed other suitable remedies, with considerable temporary relief. For the last three days, she had taken no medicine. A fever came on every day about noon, and lasted until late at night: considerable pain in the left side of the chest, and great tenderness in the cartilages of the ribs: cough incessant, without expectoration: pulse 120, small and weak. Mercurial and other purgatives were prescribed; cups applied to the chest, followed by a blister.

We shall not pursue the detail of the symptoms, or of the treatment. The pain in the chest was removed, and in the cartilages of the ribs alleviated, and the fever lost its regular remittent character; but the condition of the patient was in no way improved.

The cough continued so harrasing, that her friends supposed phthisis to be fully developed: the skin remained hot, although the patient was bathed night and day in a profuse perspiration: the tongue became clean, and of a fiery red: the strength rapidly declined: at length deafness supervened, followed after a period, by a disposition to coma, and accompanied by diarrhea. Every variety of treatment that could be suggested, had been ineffectually tried upon this person, without the satisfaction of being assured, that our prescriptions had, in a single instance, been of decided advantage. Tonics invariably did harm, in every shape, and every possible preparation of opium, was used for the purpose of mitigating the cough, without material benefit. The same remedy was equally ineffectual, in combination with astringents and absorbents, in relieving the diarrhea; it could not, however, be administered to any extent, for this latter symptom, on account of the determination to the head. Free cathartics were occasionally given, during the whole course of the disease. During the latter part of the disease, the treatment was entirely confined to alterative doses of ipecacuan and blue-mass.

Six weeks after the commencement of the disease, we met with Tate on Hysteria, and as our suspicions had been constantly directed to the suppression of the menses, as the cause of these complicated evils, the spine was immediately examined, and several of the dorsal vertebræ found extremely tender. The tartar emetic ointment was accordingly assiduously rubreb in, several times a day. In thirty-six hours, the eruption made its appearance, and the symptoms were immediately mitigated. The cough ceased as if by magic; the coma disappeared; the respiration became natural, and from this time the patient slowly and gradually recovered, without any other medicine than the occasional application of t. e. ointment, bitters, wine, and a diet accommodated to her feeble condition. Since that time, the menses have returned, and the individual has enjoyed a degree of health to which, for years before, she had been a stranger.

It is worthy of remark, that there appears to be a very general predisposition to this form of disease, in the family of this individual. Her only sister, and three of her brothers have been liable to a similar pain in the cartilages of the ribs, (combined in the case of the sister, with other symptoms of hysteria,) so much increased by sedentary pursuits, as to compel them to give up the business to which they have been educated, for more active occupations. These have been generally relieved by suitable remedies, although they have occasionally proved very troublesome. Her sister has lately had a return of this disease; when the same tenderness of the dorsal vertebræ was found to exist, and the relief obtained by the eruptive ointment was strikingly contrasted with the tedious operation of the remedies resorted to on former occasions.

*Case II.* In July last, I was called upon to prescribe for a young lady, about 21 years of age, who had been long liable to a severe pain in the region of the heart. She informed me, that there had scarcely been a cessation of the pain for the last six years; and that it had occasionally been so excruciating, as to call for the most active treatment. She had been repeatedly bled to such an extent, as seriously to impair her general health: the most rigid diet was imposed, and blisters and eruptive plasters without number, applied over the seat of the pain, all to no purpose, the disease continuing, in spite of the remedies, and at length, gradually subsiding, without any apparent cause.—On the present occasion, the paroxysm was accompanied by a slight attack of remittent fever, and the patient was unwilling to submit to any other remedial measures, than bleeding and active cathartics. Under this treatment, the pain returned to its former condition, and I frequently heard of its existence, without being requested to prescribe for its removal. During the last month, I was again requested to visit the same person, laboring under an attack of the influenza. After the symptoms of this disease were subdued, I embraced the opportunity of examining the condition of the spine, and found considerable tenterness, extending from the second to the eighth dorsal vertebra. An eruptive plaster, applied over the seat of the tenderness, produced a degree of relief, which she had not before experienced for years. A second plaster was applied, when the eruption healed, and a course of aloetic pills prescribed. She is now perfectly relieved, with every prospect of a permanent cure.

*Case III.* Mrs. B. about 23 years of age, of a florid complexion, and full habits, married about two years, has been affected since her marriage with dysmenorrhea, to a most distressing extent, accompanied with many of those nervous affections which accompany this class of diseases. In August, she was confined with an attack of bilious fever, which yielded to the customary remedies, in two or three days. She was left, however, with severe headache, continuing through the greater part of the day, with an occasional disposition to faint, while the pulse retained its fulness and strength. This last symptom led me to suspect the connexion of the disease with derangement of the sexual evacuations—the spine was examined, and the dorsal vertebræ found very tender. The spine was rubbed with the tartar emetic ointment, and the bowels kept open by the pills composed of equal portions of aloes and blue mass. The headache was almost immediately relieved on the appearance of the eruption; the uterine functions were soon after restored, and she has since enjoyed a very comfortable degree of health.

*Case IV.* Mrs. F., a lady of delicate habits, and feeble health, was confined with her first child, on the 23th December. For the first week, her condition was very favorable; but on the fifth day from her confinement, the lochia was suddenly suspended, and on the day following, symptoms of mental aberration supervened, the mind at once seeming to lose every faculty, and to be oppressed with some undefined terror and anxiety.— The pulse not much affected; the tongue covered with a thick fur. About two years before, she had suffered from an affection of the liver, which had never been perfectly relieved, and during her pregnancy, her health had been frequently interrupted in a manner that had been only relieved by active purgatives, and she had frequently complained of a fixed, dull pain, in the course of the ascending colon. This pain now returned, and with the other circumstances just mentioned, induced us to suspect that the present symptoms were owing to fœcal accumulation. Accordingly, the bowels were freely evacuated, by pills of calomel jalap and aloes, and this condition kept up for ten days. About this time, the sudden variation of the pulse, from 70 to 120, without any apparent cause, combined with the peculiarity of the mental affection, led us to suspect hysteria,—the spine was examined, and several of the dorsal vertebræ found tender upon pressure. An eruptive plaster was applied, and with the appearance of the eruption, the obliquity of the mental faculties was removed, and by great care, and strict attention to to the alvine evacuations, the patient was speedily restored to health.

It would be too much, perhaps, to affirm positively, that the symptoms, in the present instance, arose from spinal irritation; yet there is great reason to suspect, that there was a connexion, more or less intimate, between them.

Although the mental illusion was slight, and the symptoms comparatively moderate, this case certainly bore considerable resemblance to puerperal mania; and as the pathology of this disease is frequently very obscure, and the indications of treatment very embarrassing and uncertain, would not the condition of the spine be at all times worthy the attention of the profession? The influence of many forms of visceral disease upon the intellectual faculties, is one of the most familiar facts in the science of medicine. It is rational to believe, nor is there wanting evidence to confirm the fact, that the derangement of function and of sensibility in the same organs, produced by irritation of the nervous trunks by which they are supplied, will produce the same effect.

Dr. Tate has certainly erred in asserting, that deranged or suppressed menstruation is an invariable accompaniment of hysteria. We see it, on the contrary, frequently during pregnancy,—many of its symptoms very often attend lactation when the demands of the child are disproportionate to the health and strength of the mother,—nothing is more common than to meet with it, especially among those who have never borne children, long after this secretion has ceased from the decline of life, and we suspect also, that it occasionally prevails as a symptom of fluor albuss and prolapsus uteri. The general principle, however, undoubtedly holds good, and is even confirmed by the exceptions which may be offered to its literal construction.

